# Bolus Alba in Cholera

**Published:** 1914-11

**Authors:** 


**Bolus Alba in Cholera.** Julius Stumpf, Wurzburg, Munch, Med. Woch., 1914, No. 14. 200 grams are given at a dose, stirred up with water, doses being repeated almost ad lib. Of 51 cases, 47 recovered, the fatal cases being moribund.

